# Limited benefits of neoadjuvant therapy on R0 resection rate and overall survival in patients with resectable or locally advanced PDAC in randomized controlled trials

**DOI:** 10.3389/fmed.2026.1718537

**Published:** 2026-05-13

**Authors:** Hao Liang, Ya-Kun Wu

**Affiliations:** Department of Hepatobiliary Surgery, Suining Central Hospital, Suining, Sichuan Province, China

**Keywords:** borderline resectable, neoadjuvant chemotherapy, pancreatic ductal adenocarcinoma, Radiomics analysis, randomized controlled trials, resection rate, tumor microenvironment

## Abstract

A review of randomized controlled trials (RCTs) was conducted to assess the efficacy of neoadjuvant therapy for pancreatic ductal adenocarcinoma (PDAC). In this meta-analysis, neoadjuvant therapy was associated with a modest improvement in the R0 resection rate in patients with resectable PDAC (OR = 1.79, 95% CI: 1.13–2.83, *p* = 0.01), irrespective of the specific neoadjuvant chemotherapy regimen employed. However, among a specific subset of patients, the application of radiomics analysis before neoadjuvant chemotherapy may facilitate further improvements in R0 resection rates in resectable PDAC. Differences in tumor microenvironments may underlie variations in radiomic features and differential responses to neoadjuvant therapy. In the meta-analysis, neoadjuvant therapy such as the regimen with oxaliplatin, irinotecan, folinic acid, and fluorouracil (FOLFIRINOX) significantly improved the R0 resection rate (OR = 9.34, 95% CI = 4.05–21.50, *p* < 0.00001) and prognosis in patients with borderline resectable PDAC. However, the toxicity of the FOLFIRINOX regimen should be acknowledged. More phase III RCTs should be conducted to assess the efficacy of neoadjuvant chemotherapy accurately. The development of targeted immunotherapy, radiomics analysis, novel vascular resection methods, and denervation treatments may further improve the prognosis of patients with PDAC.

## 1 Background

The incidence of pancreatic ductal adenocarcinoma (PDAC) has been rising by approximately 1.0% annually, and PDAC will become the second cause of cancer-related mortality by 2030 (1). According to previous official reports, PDAC has the lowest 5-year relative survival rate among all major cancer types, at only 10% (2). Despite this alarming prognosis, effective screening programs for the primary prevention of PDAC remain lacking in clinical practice, and there are currently no established policies to support its early diagnosis.

Surgical resection remains the most effective treatment modality, with evidence of improved overall survival (OS) according to the NCCN guidelines (3). However, nearly 50% of patients present with advanced disease at the time of diagnosis (2), rendering them ineligible for curative surgery. For these patients with advanced disease, neoadjuvant therapies may help control rapid tumor progression, induce tumor downstaging and more node-negative disease, and improve pathologic response, including the margin-negative resection (R0 resection) ultimately (4). In addition, neoadjuvant chemotherapy, along with surgical resection, appears to further improve overall survival (OS) in patients with PDAC (4, 5). Thus, the utilization of neoadjuvant therapy in PDAC patients in the United States increased significantly, from 3.5% in 2004 to 26.4% in 2016 (6).

Current clinical guidelines classify PDAC into three categories based on resectability: resectable, borderline resectable, and locally advanced (7). Some recent studies have investigated the role of neoadjuvant chemotherapy across the three types of PDAC patients. Nevertheless, phase II trials with relatively small sample sizes yielded different conclusions. There remains considerable debate regarding both the indication for neoadjuvant therapy and the optimal chemotherapeutic regimen. Thus, some high-level evidence from well-designed randomized controlled trials (RCTs) should be concluded and analyzed to clarify these uncertainties and guide clinical decision-making (8). Importantly, it is crucial to identify potential reasons why some PDAC patients fail to achieve a high conversion-to-resection rate despite receiving neoadjuvant chemotherapy in these trials and to propose potential solutions to address these problems further.

In this review, we summarized and evaluated the efficacy of different neoadjuvant therapy regimens in patients with resectable, borderline resectable, and locally advanced PDAC.

## 2 Methods and materials

### 2.1 Study design and search strategy

This study followed the Preferred Reporting Items for Systematic Reviews and Meta-Analyses (PRISMA) reporting guidelines. Two researchers independently searched four databases (PubMed, Embase, the Cochrane Library, and Web of Science) on 9 October 2023 to screen articles published between 1 January 2014 and 1 September 2023. The review protocol, search strategy, inclusion and exclusion criteria, risk of bias assessment, and data synthesis plan were defined. Disagreements between the two researchers were resolved by discussion. All studies were screened by examining titles and abstracts, followed by full-text review. Duplicate studies were removed.

### 2.2 Inclusion and exclusion criteria and data extraction

To be included, studies had to meet the following criteria: (1) patients diagnosed with PDAC, (2) studies aiming to assess the efficacy of neoadjuvant therapy, (3) the resectability of pancreatic lesions was clearly defined, (4) the study design was an RCT, and (5) studies were published in English. The criteria for exclusion were (1) studies that enrolled mixed patient populations without clear subgroup definitions, (2) studies without sufficient data to extract the R0 resection or OS, and (3) single-arm trials, meeting abstracts, or studies without full text.

The following data were extracted, including the authors, year of publication, sample size, regimens of neoadjuvant therapy, dose of radiotherapy, resection rate, R0 rate, adjuvant therapy, OS, and related materials. Two researchers independently checked the data. The unavailable data were marked as ‘not available’ in the corresponding tables.

### 2.3 Statistical analysis

The Review Manager (Version 5.4) and Adobe Illustrator 2022 were used to perform meta-analysis and draw the figures. The nominal variables were presented as frequencies and proportions, and the continuous variables were presented as medians and interquartile ranges (Q_1_ and Q_3_) in the descriptive statistics. The R0 rate and OS were recorded and pooled separately in patients with resectable, borderline resectable, and locally advanced PDAC. The odds ratio (OR) and corresponding 95% confidence intervals (CI) were used as the effect measure in the meta-analysis according to the inverse variance method. The heterogeneity of the included studies was assessed using the I^2^ test. The fixed-effects model (Mantel–Haenszel method) was used to calculate pooled estimates when no statistical evidence of heterogeneity was observed (I^2^ ≤ 50%); otherwise, the random-effects model was used to calculate pooled estimates. Publication bias was visually assessed using these funnel plots. Statistical significance was considered as a *p*-value of <0.05.

## 3 Differential benefits of neoadjuvant therapy across stages of PDAC

### 3.1 Limited benefits of neoadjuvant chemotherapy in improving the R0 resection rate in patients with resectable PDAC

**Figure 1:**
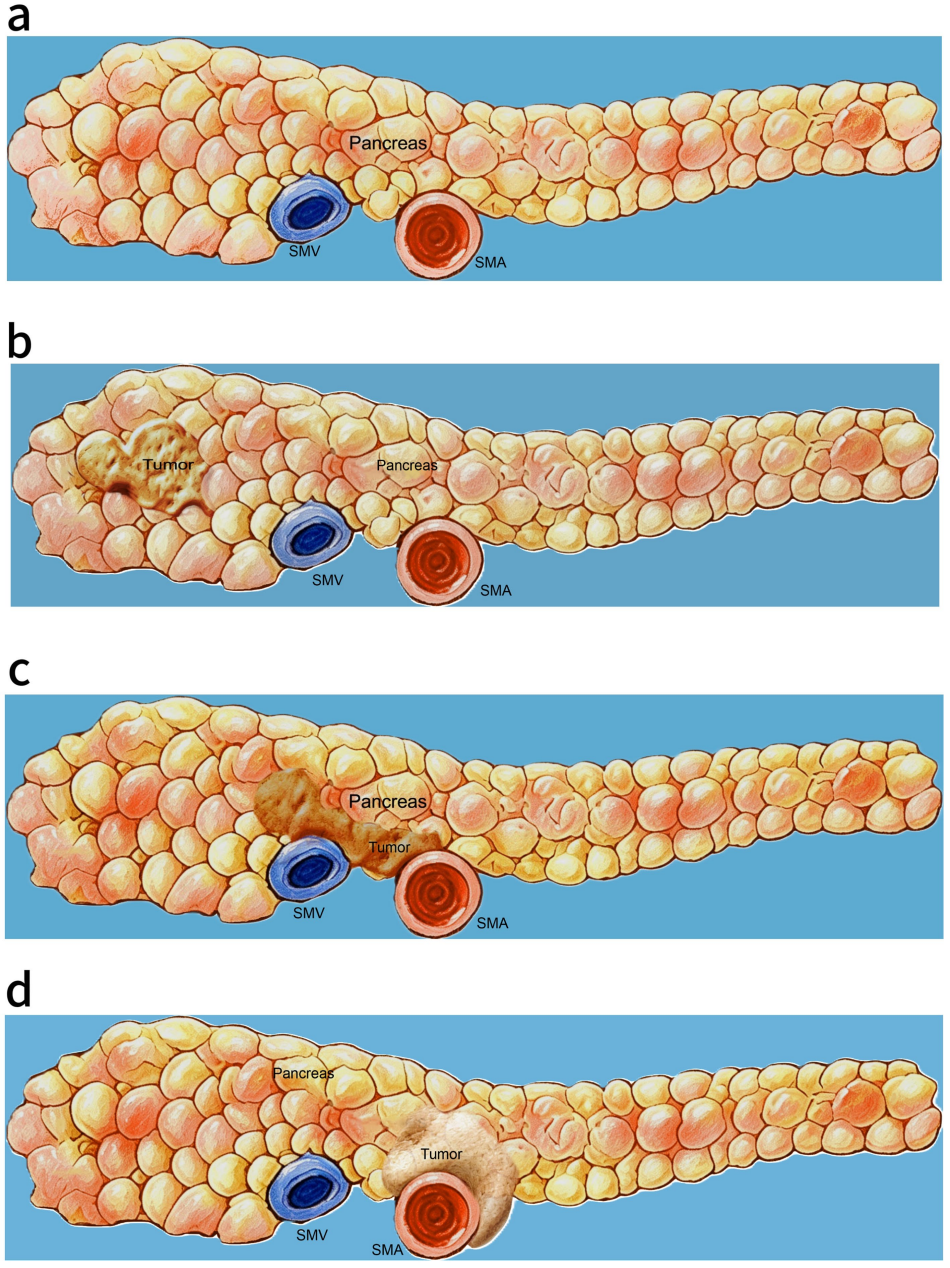
The resectability of PDAC according to the vascular involvement criteria: **(a)** Normal pancreas and SMA and SMV, **(b)** Resectable PDAC: no SMA contact of pancreatic lesions, **(c)** Borderline resectable PDAC: limited arterial contact of tumor, including the SMA ≤ 180° or the SMV ≥ 180°, and **(d)** Unresectable PDAC: arterial contact of tumor, including the SMA ≥ 180°. PDAC, pancreatic ductal adenocarcinoma; SMA, superior mesenteric artery; SMV, superior mesenteric vein.

In clinical practice, the resectability of pancreatic lesions was mainly defined according to the imaging characteristics of the tumor. Resectable PDAC is defined as a tumor that has no arterial involvement, including the celiac axis (Figure 1), superior mesenteric artery (SMA), or common hepatic artery (CHA), as well as no contact with the superior mesenteric vein (SMV) or portal vein (PV) or ≤180° venous contact without vein contour irregularities (7). However, despite preoperative classification as resectable, approximately 15% of tumors are found to be unresectable during surgery, with many tumors ultimately deemed borderline resectable or locally advanced lesions (9, 10).

This discrepancy has prompted growing interest in neoadjuvant chemotherapy for patients with resectable PDAC. Recently, several RCTs with relatively small sample sizes have been conducted to evaluate the role of neoadjuvant chemotherapy in this population. Neoadjuvant chemotherapy is often considered for patients with resectable PDAC who present with high carbohydrate antigen 19–9 (CA-199) levels, large tumor size, or significant symptoms such as severe pain (7). Nevertheless, robust clinical evidence supporting the routine use of neoadjuvant chemotherapy in these subgroups remains limited (4). To better understand the current landscape, we analyzed those recent RCTs focusing on neoadjuvant strategies in patients with resectable PDAC (Table 1).

**Table 1:** The effects data of neoadjuvant therapy or immediate surgery for resectable PDAC in the randomized controlled trials.

Data source	Regimens	Radiotherapy (Gy)	Resection rate*	R0 rate^#^ (NAT vs. IS group)	Adjuvant therapy	OS^#^ (months) (NAT vs. IS group)
Golcher et al. (13)	Gemcitabine plus Cisplatin	55.8	19/33 (57%)	52% vs. 48%	Gemcitabine	25.0 vs. 18.9
Casadei et al. (12)	Gemcitabine	45	11/18 (61.1%)	38.9% vs. 25%	Gemcitabine	22.4 vs. 19.5
Reni et al. (11)	PEXG	No	27/32 (84.4%)	63% vs. 37%	PEXG	38.2 vs. 26.4
Versteijne et al. (15)	Gemcitabine	Yes	44/65 (68%)	66% vs. 59%	Gemcitabine	14.6 vs. 15.6
Seufferlein et al. (14)	Gemcitabine plus Nab-paclitaxel	No	41/59 (69.5%)	87.8% vs. 67.4%	Gemcitabine plus Nab-paclitaxel	25.5 vs. 16.7

The difference of resection rate, R0 rate, and OS between neoadjuvant therapy groups and immediate surgery groups were not significant. *The resection rate of neoadjuvant therapy groups. ^#^The R0 rate and OS of neoadjuvant therapy groups and immediate surgery groups, respectively. PDAC, pancreas ductal adenocarcinoma; PEXG, regimens with cisplatin, epirubicin, gemcitabine and capecitabine; R0 rate, marginal negative resection rate; OS, overall survival; NAT, neoadjuvant therapy; IS, immediate surgery.

In an open-label trial, 32 patients received three cycles of cisplatin, epirubicin, gemcitabine, and capecitabine (PEXG) neoadjuvant chemotherapy (11). Approximately 85% of patients (27/32) successfully underwent surgical resection, with an R0 resection rate of 63%. The 3-year OS was 55% in those patients who received surgical resection. The PEXG neoadjuvant chemotherapy regimen appeared to improve median OS (38.2 vs. 26.4 months) and R0 resection rate (63% vs. 37%), but the differences did not reach statistical significance (*p* > 0.05).

When radiotherapy was combined with neoadjuvant chemotherapy in patients with resectable PDAC, no significant clinical benefits were observed. In a single-center trial, a total of 18 patients received two cycles of gemcitabine neoadjuvant chemotherapy and 45 Gy radiotherapy (12). Only 72.2% (13/18) of these patients proceeded to surgical resection, significantly lower than the 100% resection rate in the upfront surgery group (*p* = 0.017). The R0 resection rate was 38.9%, which was not statistically different from that of the surgery-alone group (25%, *p* = 0.489). The median OS was similar in the upfront surgery group and the neoadjuvant chemoradiotherapy group (22.4 vs. 19.5 months, *p* = 0.973). Similarly, in a multicenter trial (13), 33 patients received neoadjuvant chemoradiotherapy (gemcitabine, cisplatin, and a median radiation dose of 55.8 Gy). Approximately 57% (19/33) of patients underwent subsequent surgery. This regimen of chemoradiotherapy did not significantly improve the R0 resection rate (52% vs. 48%, *p* = 0.81) or OS (25.0 vs. 18.9 months, *p* = 0.79). Importantly, this multicenter trial was prematurely terminated due to a lack of significant efficacy.

In addition, several clinical trials have investigated different neoadjuvant chemotherapy regimens with larger sample sizes to validate the potential benefits of neoadjuvant chemotherapy. In a recent randomized phase II trial in 2023 (14), a total of 59 patients received neoadjuvant chemotherapy (nab-paclitaxel and gemcitabine) combined with radiotherapy. Surgical resection was achieved in nearly 70% (41/59) of patients, with an R0 resection rate of 87.8%. The median OS was 25.5 months. However, the primary endpoint of the study was still not reached at the time of reporting. Another phase III clinical trial, including 133 patients, enrolled 65 individuals in the neoadjuvant therapy arm, who received gemcitabine-based chemotherapy and radiotherapy (15). Approximately 68% (44/65) of patients underwent resections, and the R0 resection rate was 66%. The median OS was 14.6 months in the neoadjuvant chemotherapy groups. However, the difference did not reach statistical significance (*p* > 0.05). Furthermore, in a systematic review and meta-analysis without significant heterogeneity of studies included (16), the results showed that gemcitabine-based neoadjuvant chemotherapy regimens failed to improve the OS of patients (HR = 0.76, 95% CI: 0.52–1.11).

**Figure 2:**
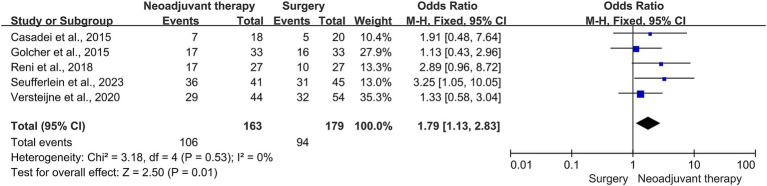
Forest plot for the R_0_ resection rate between the neoadjuvant therapy groups and upfront surgery groups in patients with resectable PDAC. R_0_, marginal negative resection; PDAC, pancreatic ductal adenocarcinoma.

**Figure 3:**
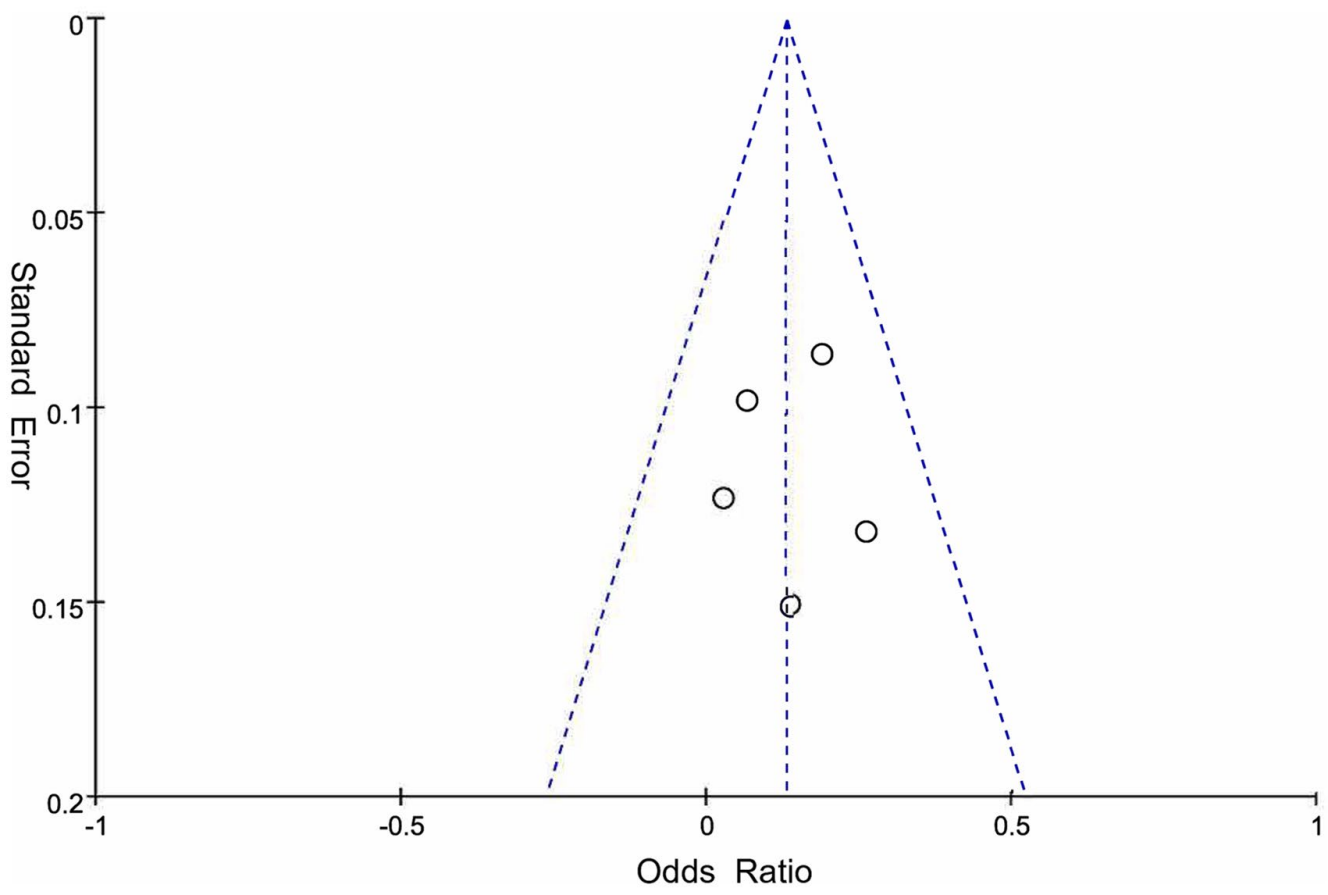
Funnel plot for these studies on the efficacy of neoadjuvant therapy in patients with resectable PDAC. PDAC, pancreatic ductal adenocarcinoma.

**Figure 4:**
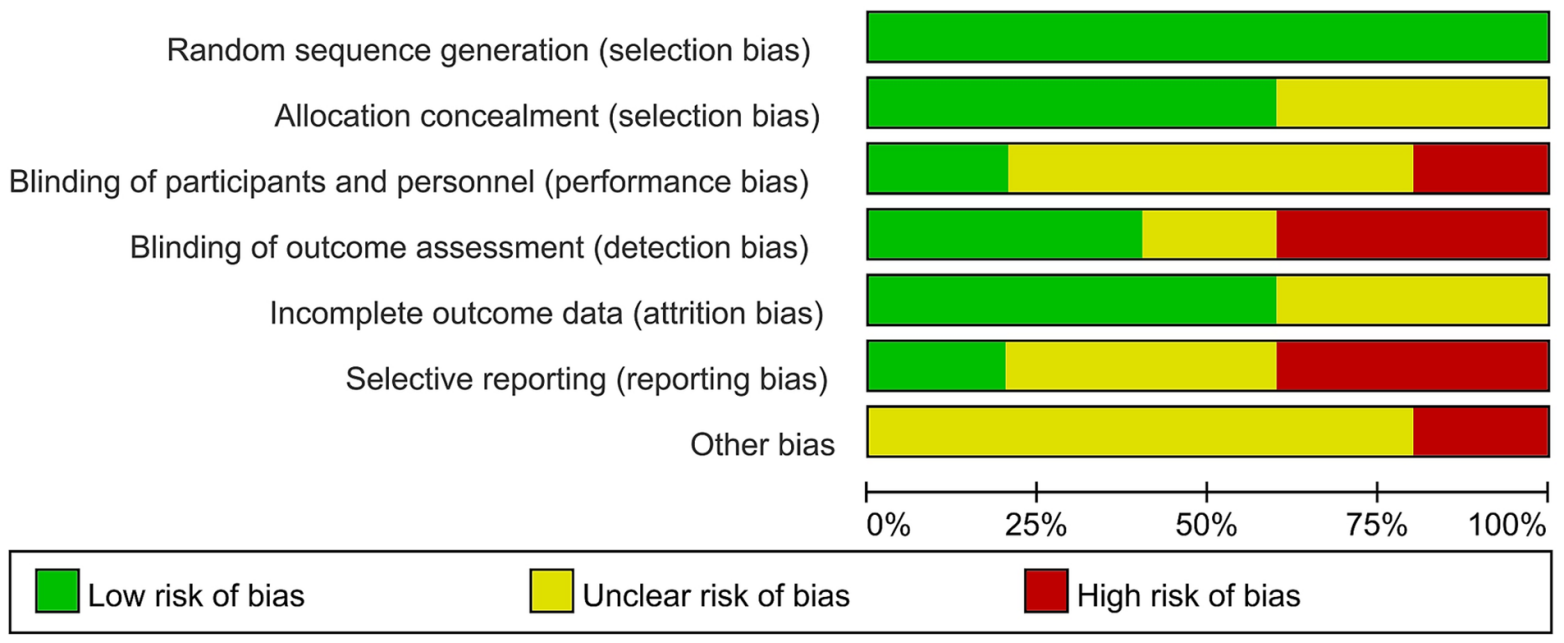
Risk of bias graph for these studies on the efficacy of neoadjuvant therapy in patients with resectable PDAC. PDAC, pancreatic ductal adenocarcinoma.

This meta-analysis included 163 patients who received neoadjuvant therapy and 179 patients who received surgical treatment. The R0 resection rate was 65.0% (106/163) and 52.5% (94/179) in the neoadjuvant therapy and surgery alone groups, respectively. These neoadjuvant chemoradiotherapy treatments have shown a limited impact on improving the R0 resection rate in patients with resectable PDAC (OR = 1.79, 95% CI = 1.13–2.83, *p* = 0.01), as shown in Figure 2. The heterogeneity across these five studies was not significant (I^2^ = 0, *p* = 0.53), which enhanced the reliability of the meta-analysis results. There was publication bias in the funnel plot; however, the symmetric distribution of studies suggests that some studies with negative results were hidden (Figure 3). This figure supported the robustness of the results of the meta-analysis. The risk of bias of those included studies is shown in Figure 4. The primary bias was the performance bias, which was a common limitation in surgical treatments. The data revealed that the methodological quality of the included studies was moderate to high, supporting the validity of the results of the meta-analysis.

To improve the R0 resection rate and the OS of patients with resectable PDAC, selecting an optimal neoadjuvant chemotherapy regimen is of critical importance. However, evidence has suggested that resectable PDAC patients were unlikely to benefit from the gemcitabine-based neoadjuvant chemotherapy regimens. Consequently, these alternative neoadjuvant strategies, including combinations of gemcitabine with erlotinib (17), granulocyte-macrophage colony-stimulating factor, and allogeneic PDAC vaccine (18), were explored. Despite these efforts, high-quality phase III RCTs remain scarce. This gap might stem from insufficient consideration of some key biological heterogeneities, such as tumor microenvironment or geographical or ethnic variations among resectable PDAC patients (19), or from the lack of standardized metrics to quantify such heterogeneities of lesions. Moreover, a subset of PDAC lesions initially classified as resectable ultimately proved to be unresectable lesions at surgery after receiving the neoadjuvant therapy (20). For these patients, the current efficacy of neoadjuvant therapy remains suboptimal and warrants further investigation.

### 3.2 Neoadjuvant chemotherapy improves R0 resection rates and prognosis in patients with borderline resectable PDAC

Borderline resectable PDAC is defined as a tumor with limited arterial contact (Figure 1), including the celiac axis, SMA, or CHA with ≤180° involvement, or tumor contact with the SMV, PV, or inferior vena cava (7). In clinical practice, a significant number of patients initially diagnosed with borderline resectable PDAC are ultimately found to be unresectable or locally advanced at the time of surgery. Surgery, including the vascular resections and reconstructions, remains the treatment end-goal option for patients with borderline resectable PDAC (21). To increase the likelihood of achieving R0 resection and to facilitate successful conversion therapy, neoadjuvant chemotherapy has emerged as a promising treatment strategy for patients with borderline resectable PDAC. Consequently, few clinical trials have been conducted to assess the efficacy of neoadjuvant chemotherapy in improving clinical outcomes for individuals with borderline resectable PDAC (Table 2).

**Table 2:** The effects data of neoadjuvant therapy or immediate surgery for borderline resectable PDAC in the randomized controlled trials.

Data source	Regimens	Radiotherapy (Gy)	Resection rate^†^	R0 rate^#^ (NAT vs. IS group)	Adjuvant therapy	OS (months)^#^ (NAT vs. IS group)
Jang et al. (23)	Gemcitabine	45	17/27 (62.9%)	82.4% vs. 33.3%*	Gemcitabine	21 vs. 12*
Versteijne et al. (15)	Gemcitabine	Yes	28/54 (52%)	79% vs. 13%*	Gemcitabine	17.6 vs. 13.2*
Ghaneh et al. (22)	FOLFIRINOX	No	11/20 (55%)	18.2% vs. 14.3%	N. A	84% vs. 39%^&^*

^#^The R0 rate and OS of neoadjuvant therapy group and immediate surgery group, respectively. ^†^The resection rate of neoadjuvant therapy group. ^&^1-year OS. *p < 0.05. PDAC, pancreas ductal adenocarcinoma; FOLFIRINOX, regimens with oxaliplatin, irinotecan, folinic acid and fluorouracil; R0 rate, marginal negative resection rate; OS, overall survival; N.A, not available; NAT, neoadjuvant therapy; IS, immediate surgery.

The neoadjuvant chemotherapy may provide clinical benefits for patients with borderline resectable PDAC, regardless of whether neoadjuvant radiotherapy is prescribed (4). In an open-label clinical trial (22), the FOLFIRINOX (oxaliplatin, irinotecan, folinic acid, and fluorouracil) neoadjuvant chemotherapy regimen was prescribed. Nearly 55% (11/20) of patients were eligible for the subsequent surgical resection, with the R0 resection rate of 18.2% (2 out of 11) in this subgroup. Importantly, patients who received neoadjuvant chemotherapy exhibited improved 1-year OS (84% for the neoadjuvant chemotherapy group, 60% for the neoadjuvant chemoradiotherapy group, and 39% for the upfront surgery group). The difference in survival rates was statistically significant (*p* = 0.0028).

In addition, radiotherapy combined with chemotherapy has been shown to further improve clinical outcomes in patients with borderline resectable PDAC. In an open-label trial (23), neoadjuvant chemoradiotherapy (gemcitabine combined with 45 Gy radiation therapy) was prescribed to 26 (86.7%) patients with borderline resectable PDAC. Of these, 17 (62.9%) patients subsequently underwent surgical resection, with a high R0 resection rate of 82.3% (14/17). The median OS was 21 months, significantly longer than that of the upfront surgery group (12 months, *p* = 0.028). Similarly, in a large-sample trial involving 113 borderline resectable PDAC cases (15), a total of 54 patients received neoadjuvant chemoradiation (gemcitabine and radiotherapy). Among them, 52% (28/54) of patients were able to undergo surgical resection, and the R0 resection rate reached 79% (22/28). The median OS was 17.6 months in this cohort, which was also significantly higher than that of the upfront surgery group (13.2 months, *p* = 0.029). These findings support the current mainstream view that neoadjuvant chemoradiotherapy offers a significant advantage for patients with borderline resectable PDAC (5).

**Figure 5:**
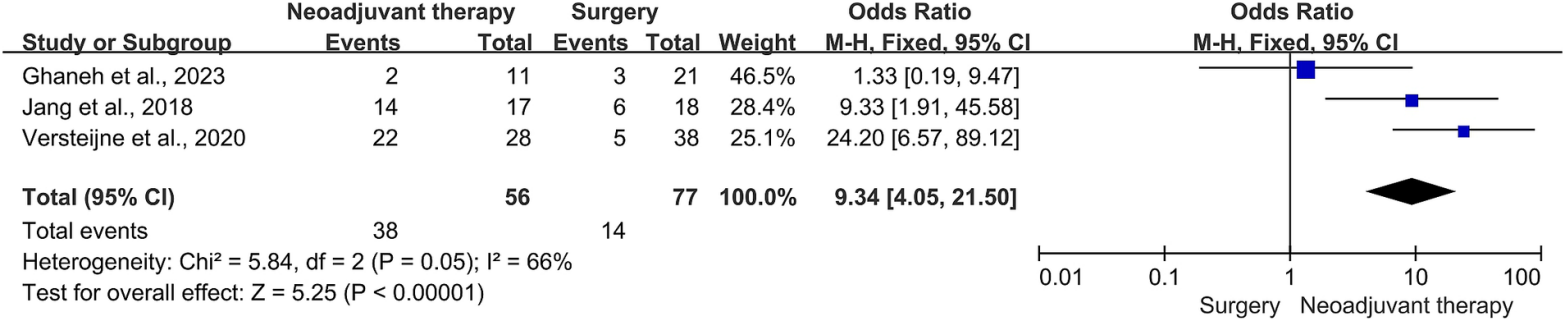
Forest plot for R_0_ resection rate between the neoadjuvant therapy groups and upfront surgery groups in patients with borderline resectable PDAC. R_0_, marginal negative resection; PDAC, pancreatic ductal adenocarcinoma.

**Figure 6:**
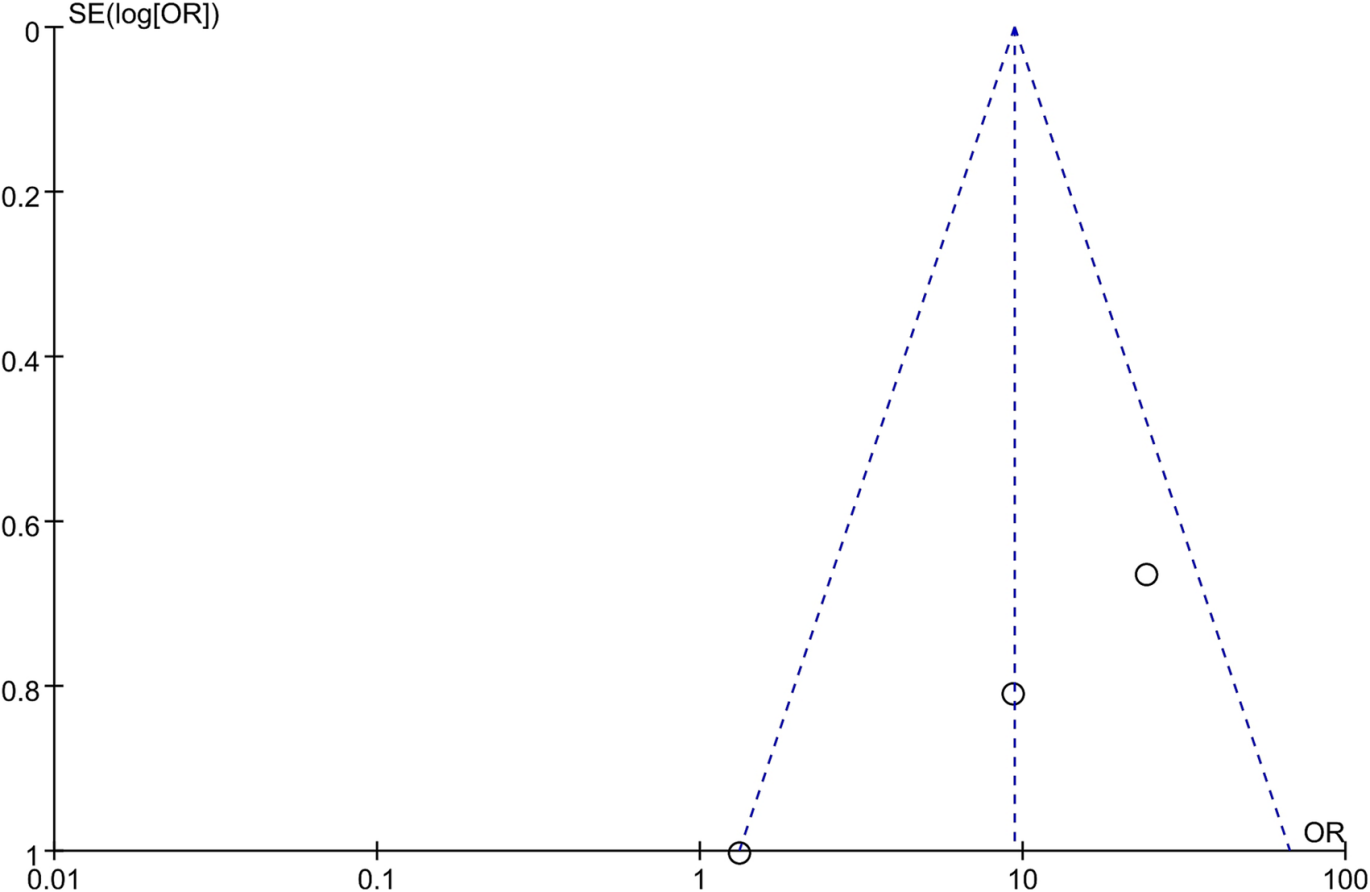
Funnel plot for these studies on the efficacy of neoadjuvant therapy in patients with borderline resectable PDAC. PDAC, pancreatic ductal adenocarcinoma.

**Figure 7:**
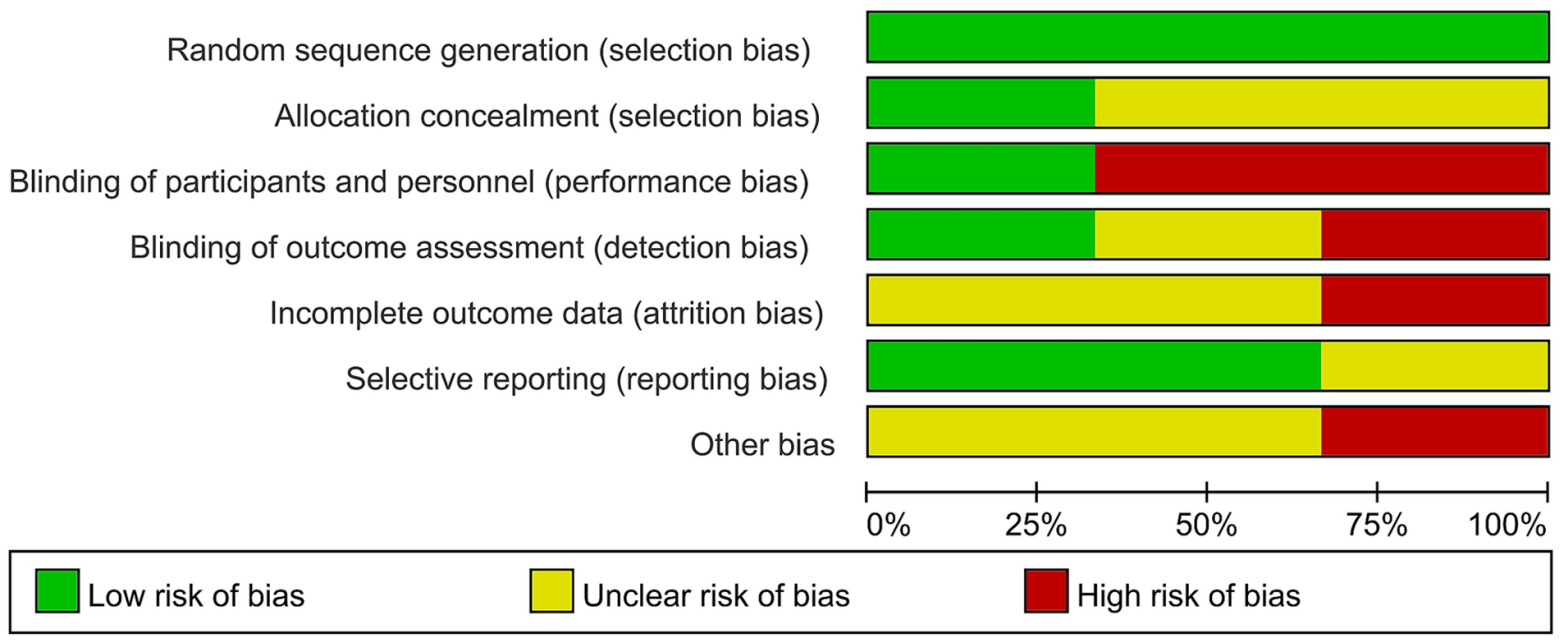
Risk of bias graph for these studies on the efficacy of neoadjuvant therapy in patients with borderline resectable PDAC. PDAC, pancreatic ductal adenocarcinoma.

This meta-analysis included three studies, with a total of 56 patients who received neoadjuvant therapy and 77 patients who underwent surgical resection. The R0 resection rate was 67.8% (38/56) and 18.2% (14/77) in the neoadjuvant therapy group and surgery alone group, respectively. These neoadjuvant therapies have shown a positive impact on improving the R0 resection rate in patients with borderline resectable PDAC (OR = 9.34, 95% CI = 4.05–21.50, *p* < 0.00001), as shown in Figure 5. The heterogeneity across these studies was not significant (I^2^ = 66%, *p* = 0.05), supporting the reliability of the result that neoadjuvant therapies improved the R0 resection rate of patients with borderline resectable PDAC. The funnel plot was asymmetric (Figure 6), which showed some publication bias or small-study effects in those studies. No selection bias from those studies was identified, and the risk of bias from the included studies is shown in Figure 7. In another systematic review and patient-level meta-analysis of FOLFIRINOX-based neoadjuvant therapy, the pooled resection rate was 67.8%, the R0 resection rate was 83.9%, and the median OS was 22.2 months (24), which supported the use of neoadjuvant therapy in patients with borderline resectable PDAC. FOLFIRINOX is a commonly used regimen for borderline resectable PDAC.

In light of the significant efficacy and manageable toxicity profile of certain agents, an increasing number of studies have begun to evaluate the FOLFIRINOX-based neoadjuvant chemotherapy regimen for borderline resectable PDAC. A previous study involving 51 patients compared neoadjuvant treatment either with FOLFIRINOX or gemcitabine with nab-paclitaxel (25). No significant difference in the R0 resection rate, OS, and major complications was observed in the two groups. Notably, modified FOLFIRINOX regimens demonstrated promising efficacy (26). A recent study evaluated the efficacy of the four-cycle modified FOLFIRINOX regimen (mFOLFIRINO: oxaliplatin, irinotecan, fluorouracil, and leucovorin) treatment (27) and reported that 49% (32/65) of patients underwent surgical resection, with an R0 resection rate of 88% (28 out of 32) among those patients. The median OS of patients receiving mFOLFIRINOX as neoadjuvant therapy was 29.8 (95% CI: 21–36.6) months, indicating substantial clinical benefits.

In addition to the FOLFIRINOX regimen, several other neoadjuvant strategies have been investigated, including the combination of gemcitabine with S1 (28), FOLFIRINOX and intensity-modulated radiation therapy concurrent with fixed-dose rate-gemcitabine (29), gemcitabine plus nab-paclitaxel (30), and PD-1 blockade (31). However, these clinical trials are generally limited by small sample sizes, the heterogeneity of PDAC cases, and potential ethnic and regional variations among patient populations. As a result, the R0 resection rate varies widely across trials, from 40% (32) to 93.1% (33), highlighting the need for larger, well-designed multicenter trials to standardize neoadjuvant approaches and better define optimal treatment strategies.

### 3.3 Low surgical conversion rate of neoadjuvant chemotherapy in locally advanced PDAC

Localized unresectable PDAC cases mainly consist of the locally advanced lesions without non-regional lymph node metastasis and distant metastasis (7). At diagnosis, approximately 30–35% of patients present with locally advanced disease (1). The low surgical conversion rate in these patients was a significant clinical challenge. Therefore, few clinical trials have specifically evaluated the efficacy of different neoadjuvant chemotherapy regimens in this patient population (Table 3). The primary objective of these studies was to improve the resectability of locally advanced lesions by increasing the surgical conversion rate through effective neoadjuvant treatments.

**Table 3:** The effects data of different neoadjuvant therapy regimens for locally advanced PDAC in the randomized trials.

Data source	Regimens of neoadjuvant therapy	Radiotherapy (Gy)	Control set	Resection rate (NAT vs. IS group)	R0 rate (NAT vs. IS group)	OS (months) (NAT vs. IS group)
Murphy et al. (36)	FOLFIRINOX plus losartan	25 or 30	No	69.4% (34/49)	61.2% (30/49)	31.4
Philip et al. (35)	Gemcitabine plus Nab-paclitaxel	No	No	15.1% (16/106)	41% (7/17)	18.8
Kunzmann et al. (34)	Gemcitabine plus Nab-paclitaxel	No	Gemcitabine plus Nab-paclitaxel and sequential FOLFIRINOX	35.9% vs. 43.9%^#^	65% vs. 69%^#^	18.5 vs. 20.7^#^

^#^The R0 rate and OS of neoadjuvant therapy group and immediate surgery group, respectively. The difference of resection rate, R0 rate, and OS between different groups were not significant. PDAC, pancreas ductal adenocarcinoma, FOLFIRINOX, regimens with oxaliplatin, irinotecan, folinic acid and fluorouracil; R0 rate, marginal negative resection rate; OS, overall survival; NAT, neoadjuvant therapy; IS, immediate surgery.

A randomized, phase II trial (34) showed that the efficacy of the nab-paclitaxel plus gemcitabine group or the sequential FOLFIRINOX was similar in improving the R0 resection rate (65% vs. 69%, *p* = 0.99) and OS (18.5 vs. 20.7 months, *p* = 0.53). This study included 130 patients with locally advanced disease, with surgical conversion rates of 35.9% in the nab-paclitaxel plus gemcitabine group and 43.9% in the sequential FOLFIRINOX group. The difference was not statistically significant (*p* = 0.38). Another large-scale study showed that only 16% (17/107) of enrolled patients who received preoperative induction therapy with nab-paclitaxel and gemcitabine underwent surgical resection (35). The R0 resection status was achieved in seven cases (41.1%). To improve surgical conversion rates, emerging evidence has suggested that losartan may enhance the delivery of cytotoxic agents by modulating the tumor microenvironment. Thus, a single-arm clinical trial explored this potential synergistic effect (36). In this study, 49 patients received preoperative induction therapy consisting of FOLFIRINOX combined with losartan, followed by chemoradiotherapy. The results were promising: 86% of patients proceeded to surgery, and an R0 resection was achieved in 69% (34/49) of cases. The median OS rate reached 31.4 months. These findings highlight the potential of losartan as a treatment adjunct to enhance resectability in locally advanced cases. However, its role warrants further validation through large-scale RCTs.

Currently, due to limited sample sizes, some studies have combined locally advanced and borderline resectable PDAC cases in efforts to evaluate the efficacy of various neoadjuvant regimens—such as the addition of cisplatin and capecitabine to nab-paclitaxel and gemcitabine (37), and interferon-based chemoradiotherapy (38). Although these regimens show promising surgical conversion rates, their outcomes may lack stability and generalizability when applied specifically to locally advanced PDAC.

## 4 Strategies to overcome challenges associated with neoadjuvant therapy across disease stages in PDAC

### 4.1 Potential values of radiomics in predicting resectable PDAC patients receiving R0 resection

In several clinical trials, a notable proportion (30 to 50%) of patients with resectable PDAC who received neoadjuvant chemotherapy were either diagnosed with disease progression or underwent R1 resection (Table 4). This highlights a significant clinical challenge in the management of patients with initially resectable PDAC.

**Table 4:** The neglected data of PDAC patients with the failure of neoadjuvant therapy in these clinical trials.

Data source	Types of PDAC patients	Regimens	Radiotherapy (Gy)	Failure of resection number (rate)	Progressive diseases	Death number (rate)
Reni et al. (37)	Resectable	PEXG	No	3/32 (9.4%)	1/32 (3.1%)	0/32
Golcher et al. (13)	Resectable	Gemcitabine plus Cisplatin	55.8	14/33 (42%)	4/33 (12%)	0/33
Casadei et al. (12)	Resectable	Gemcitabine	45	7/18 (38.8%)	4/18 (22.2%)	1/18 (5.5%)
Seufferlein et al. (14)	Resectable	Gemcitabine plus Nab-paclitaxel	No	18/59 (30.5%)	4/59 (7%)	0/59
Versteijne et al. (15)	Resectable plus borderline resectable	Gemcitabine	Yes	19/91 (20.8%)	16/91 (17.5%)	2/91 (2.1%)
Jang et al. (23)	Borderline resectable	Gemcitabine	45	10/27 (37.0%)	2/27 (7.4%)	0/27
Ghaneh et al. (22)	Borderline resectable	FOLFIRINOX	No	9/20 (45%)	2/20 (10%)	1/20 (5%)
Murphy et al. (36)	Locally advanced	FOLFIRINOX plus losartan	25 or 30	15/49 (30.6%)	6/49 (12.2%)	0/49
Philip et al. (35)	Locally advanced	Gemcitabine plus Nab-paclitaxel	No	90/106 (84.9%)	22/106 (20.7%)	0/106
Kunzmann et al. (34)	Locally advanced	Gemcitabine plus Nab-paclitaxel	No	78/130 (60%)	11/130 (8.4%)	0/130

PDAC, pancreas ductal adenocarcinoma; PEXG, regimens with cisplatin, epirubicin, gemcitabine and capecitabine; FOLFIRINOX, regimens with oxaliplatin, irinotecan, folinic acid and fluorouracil.

In a clinical trial evaluating neoadjuvant chemoradiotherapy (gemcitabine combined with a radiotherapy regimen) for resectable PDAC cases, nearly 40% (7 out of 18) of patients ultimately became ineligible for subsequent surgical resection, with one-quarter of these cases diagnosed with disease progression (12). Notably, one patient died during the neoadjuvant treatment period. In another regimen combining gemcitabine, nab-paclitaxel, and radiotherapy, approximately 30.5% (18/59) of patients failed to proceed to surgical therapy, and 7% of them were diagnosed with progressive disease (14). Similarly, in a larger trial utilizing a neoadjuvant protocol of gemcitabine, cisplatin, and radiotherapy, approximately 42% (14 out of 33) were deemed unsuitable for surgical intervention, with nearly 15% of cases exhibiting disease progression (13). Among patients receiving the PEXG neoadjuvant chemotherapy regimen (11), approximately 5% of patients experienced local disease progression. Additionally, two patients opted to discontinue neoadjuvant therapy and proceeded directly to surgical resection.

To accurately identify patients with resectable PDAC who are candidates for R0 resection, the limitations of current radiological assessment methods for tumor staging must first be acknowledged. Conventional imaging techniques often fail to accurately determine resectability, as evidenced by the fact that up to 30% of patients deemed eligible for surgery are found to have intraoperative metastases (13). This highlights a critical shortcoming in existing radiological evaluation protocols. Emerging tools such as radiomics offer a promising solution to this challenge. By extracting and analyzing high-dimensional data from medical images, radiomics can capture subtle tumor characteristics that are not discernible through conventional visual interpretation. Computed tomography (CT)-based radiomics models could predict fibrosis in PDAC lesions and neoadjuvant chemotherapy efficacy evaluation values (39, 40). In particular, applying radiomics to visible tumor lesions before and after neoadjuvant chemotherapy may provide more accurate and objective assessments of treatment response (41). Notably, radiomics data from patients both prior to and following neoadjuvant therapy are already available. These datasets represent a valuable resource for addressing key clinical uncertainties—particularly in identifying which patient subgroups are most likely to benefit from neoadjuvant therapy.

Different radiomics features of PDAC lesions may reflect differences in tumor microenvironments (42). PDAC is characterized by significant heterogeneity in immune cell composition and fibroblasts in the tumor microenvironment (43). Neoadjuvant chemotherapy could alter the tumor microenvironment, including inhibiting the proliferation of tumor cells, upregulating multiple complement genes (C1R, C1S, and C3), increasing cancer-associated fibroblasts (44), increasing CD8 + cell density, and decreasing T regulatory cell and M2 macrophage density (45). A recent study has shown the non-invasive prediction of the tumor microenvironment via machine learning-driven CT radiomics tools, with AUC values exceeding 0.9 (46). Future studies that characterize the distinct radiomics and clinical traits of these subgroups could significantly improve patient selection, thereby enhancing the effectiveness and efficiency of neoadjuvant strategies (47).

### 4.2 Exploring an optimized and safer FOLFIRINOX regimen in borderline resectable PDAC patients

In patients with borderline resectable PDAC, neoadjuvant chemotherapy could significantly improve both the R0 resection rate and OS rate. However, severe adverse events may disrupt the whole neoadjuvant treatment procedure for PDAC and may influence the clinical outcomes directly. In a large clinical trial, approximately 18% of patients experienced at least one serious adverse event during neoadjuvant chemotherapy (22). Notably, fatal events have been reported: one patient died during gemcitabine plus capecitabine neoadjuvant therapy, and another patient died during FOLFIRINOX-based therapy.

Among the commonly used neoadjuvant regimens, FOLFIRINOX shows a high response rate, but its toxicity profile warrants close monitoring. In a previous study, half of the patients (27/54) receiving neoadjuvant gemcitabine plus radiotherapy experienced at least one serious adverse event, with two treatment-related deaths reported (15). In contrast, a patient-level meta-analysis of FOLFIRINOX-based neoadjuvant therapy showed no treatment-related deaths, but grade III–IV adverse events were not uncommon—neutropenia being the most frequent, occurring in 17.5 per 100 patients (24). Another study reported that nearly 30% of patients in the FOLFIRINOX group experienced grade III adverse events (25), which was significantly lower than the 70% observed in the gemcitabine plus nab-paclitaxel group (*p* = 0.010). Overall, existing evidence from clinical trials and meta-analyses has supported the use of FOLFIRINOX as an effective neoadjuvant regimen for PDAC. More recently, modified FOLFIRINOX (mFOLFIRINOX) has been explored to improve tolerability while maintaining efficacy. However, even with dose modifications, 57% of patients still experienced at least one grade III adverse event associated with mFOLFIRINOX (27), underscoring the need for careful toxicity management.

Adverse events associated with neoadjuvant treatment of the modifications of the FOLFIRINOX regimen cannot be entirely avoided in some patients. Furthermore, when some patients were diagnosed with a performance status over 3 points, the FOLFIRINOX regimen was not the optimal neoadjuvant regimen for them in the National Comprehensive Cancer Network (NCCN) guidelines (48). Thus, optimizing and exploring safer regimens in this patient population remains a critical priority. The KRAS oncogene has played an important role in PDAC initiation, and accumulating evidence has indicated that neural signals regulate critical functions of PDAC, such as their proliferation and dissemination (49, 50). The non-covalent and selective KRAS^G12D^ inhibitor, MRTX1133, was developed (51), and MRTX1133 may act synergistically with erlotinib in the treatment of advanced PDAC (52). As for the influence of neural signals, previous studies have revealed a correlation between nerve density and increased survival of patients receiving non-selective *β*-blockers (53). In addition, there were other factors influencing the outcomes of PDAC patients. Ethnic disparities in PDAC patients played a role in tumor-specific molecular alteration for prognosis, such as Asian patients with fewer CDKN2A mutations (54) and oriental PDAC patients, including higher mutation rates of DNA damage repair-related genes (55).

Neoadjuvant treatments for borderline resectable PDAC have met with success, and the R0 resection is important. Furthermore, recent studies have supported the view that pertains to pancreatectomy with vascular resection for patients with borderline resectable PDAC (21). The new method of vascular resection could improve the R0 resection rate, including left-to-right pancreatoduodenectomy at the splenic vessels (56). Arterial resection could be conducted in those selected patients in centers with appropriate expertise (57–59). The benefits of neoadjuvant therapy are maximized when patients with borderline resectable PDAC ultimately undergo successful R0 resection.

### 4.3 Need for larger, phase III RCTs for locally advanced PDAC patients

Patients with locally advanced PDAC who develop distant metastasis following neoadjuvant therapy are generally no longer candidates for curative surgical resection. This scenario is not uncommon and represents a significant clinical challenge. In a larger clinical trial, nearly 30% (9 out of 30) of patients did not proceed to receive exploratory laparotomy therapy due to disease progression despite completing the neoadjuvant treatment (34). Similarly, in another study, 44 PDAC patients discontinued the neoadjuvant therapy, with 18.1% (8 patients) being diagnosed with disease progression (35). These patients inevitably lose the opportunity for surgical intervention. Although their prognosis is poor and OS remains short, these patients hold substantial value for the design and enrollment of future clinical trials—particularly those evaluating novel systemic therapies or palliative strategies. However, the limited survival duration poses practical challenges for trial enrollment and longitudinal follow-up.

Notably, the current analysis is based on data from existing RCTs, which often have strict inclusion criteria. These included RCTs with relatively small sample sizes, and some of the trials were terminated in advance due to a lack of significant efficacy. In addition, the loss of information was reported. As a result, real-world PDAC patients with poorer clinical outcomes may be underrepresented.

## 5 Limitations and futures

There were several limitations in this review of RCTs. First, the majority of the included studies were phase II trials, which typically involve relatively small sample sizes, and the statistical power of these trials may be limited. Therefore, larger phase III RCTs are needed. Second, we excluded trials that enrolled mixed patient populations, such as those combining resectable and borderline resectable PDAC patients, to maintain homogeneity across subgroups. Our aim was to specifically evaluate the effects of neoadjuvant chemotherapy in distinct PDAC stages. However, the inclusion of heterogeneous populations in other studies may introduce confounding factors. Therefore, such studies were not included in this review. Third, PDAC can be broadly classified into two anatomical subtypes: pancreatic head cancer and pancreatic body/tail cancer. These subtypes may differ in terms of tumor biology, surgical accessibility, and clinical outcomes. However, the available randomized trials did not consistently report outcomes stratified by tumor location. The potential heterogeneity between these subtypes warrants further investigation in future studies.

Advances in radiomics analysis, tumor microenvironment, neural signals inhibiting the proliferation of tumor cells, and techniques for vascular resection hold great promise for transforming PDAC management. As these technologies evolve, clinicians may achieve more precise staging, earlier assessment of neoadjuvant chemotherapy response, and improved R0 resection and clinical outcomes. Currently, there are some studies using the related technologies to manage patients with PDAC, including radiomics, new methods of vascular resection, and circulating tumor DNA (ctDNA).

These radiomics signatures (60) derived from 40-keV virtual monoenergetic images had exhibited exceptional performance in distinguishing early from advanced stages in PDAC (area under the receiver operating characteristic curves [AUCs] of 0.94 in the test cohorts). Analysis of tumor-related radiomics features (61) improves preoperative assessment of tumor involvement of the superior mesenteric artery in patients with PDAC, with better performance compared with the radiologist assessment (AUCs, 0.71 vs. 0.54; *p* < 0.001). The recently developed artificial intelligence model could allow automatic quantification of vascular involvement and classification of resectability for PDAC (62). In addition, these CT-based radiomics signatures may help preoperatively predict nodal positivity in patients with PDAC (63). A radiomics-clinical nomogram of PDAC could predict 1-year recurrence after radical resection, with AUCs of 0.764 in the validation set (64). In the future, the potential application of radiomics on other structures may enable a comprehensive preoperative assessment of resectability. For PDAC patients with vascular involvement, neoadjuvant treatment followed by pancreatectomy with vascular resection could be proposed as a curative option (65). However, vascular resection may be associated with higher 90-day mortality rates (66). Thus, careful consideration is essential in determining the need for vascular resection.

As for the ctDNA, a recent prospective study (67) has reported that 227 (31%) patients underwent subsequent resection, with significantly fewer mutant KRAS-positive ctDNA patients undergoing resection (11% vs. 34%; *p* < 0.001). Additionally, patients in the ctDNA-positive group (68) had a significantly higher frequency of occult metastases than those of patients in the ctDNA-negative group (51% vs. 20%, *p* < 0.001). Positive ctDNA was significantly associated with shorter median disease-free survival (69) during the surveillance period (11.40 months for ctDNA-positive vs. not recorded for ctDNA-negative; HR = 12.38, *p* < 0.0001). A meta-analysis has supported the utility of preoperative KRAS-mutated ctDNA testing as a prognostic marker for resected PDAC (70).

**Figure 8:**
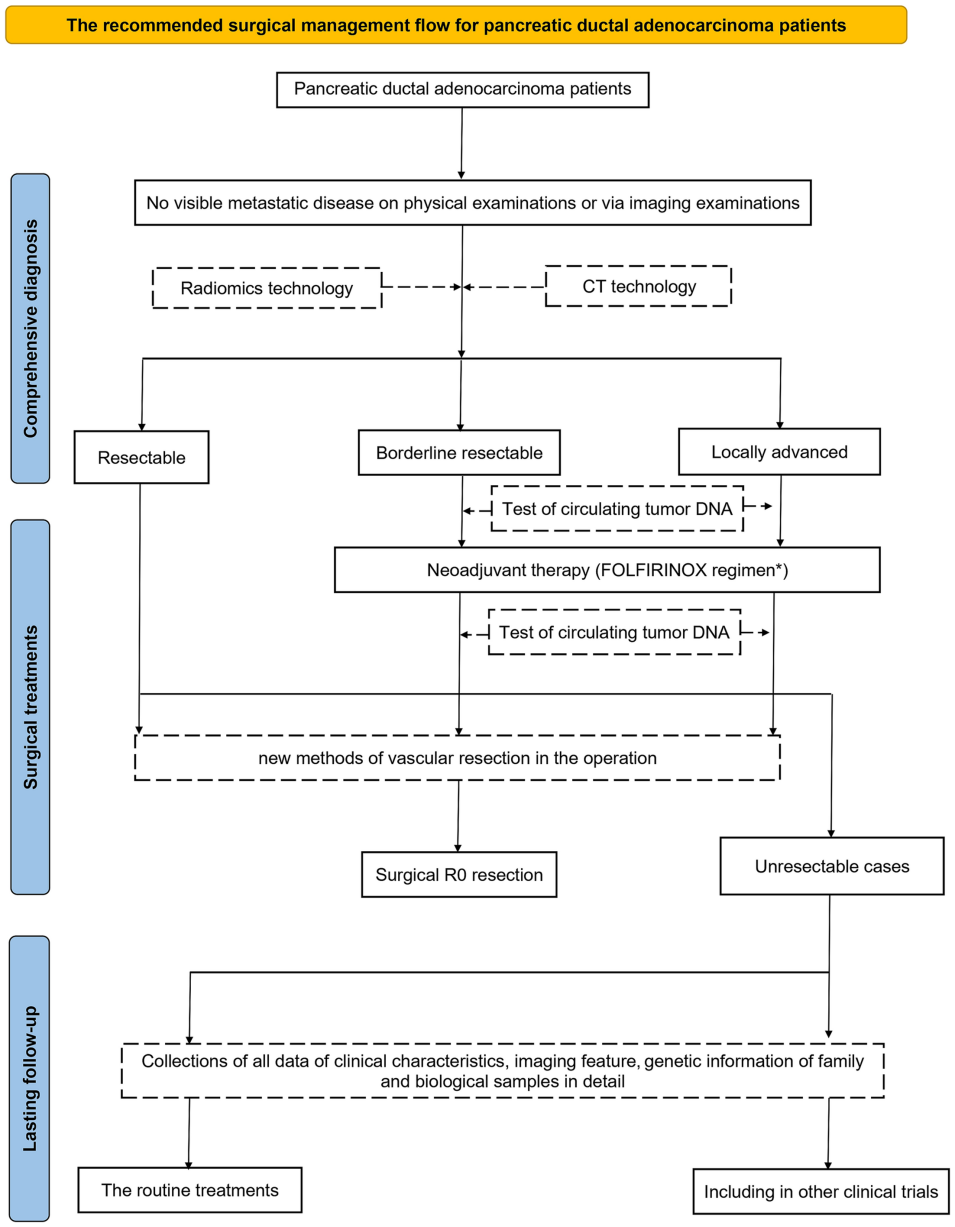
Potential additional pathways in the future management algorithm for patients with PDAC. FOLFIRINOX, regimens with oxaliplatin, irinotecan, folinic acid, and fluorouracil; R0, marginal negative resection. The extra potential effective steps for improving the prognosis of patients.

While significant challenges remain, integrating these innovative tools into future clinical trials will be crucial for optimizing personalized treatment strategies in PDAC (Figure 8). Although the path forward is complex, the potential benefits for PDAC patients make this endeavor well worth pursuing.

## 6 Conclusion

The efficacy of neoadjuvant chemotherapy in patients with resectable or locally advanced PDAC remains limited. However, radiomics analysis prior to neoadjuvant treatment may help identify subgroups of resectable PDAC patients who are more likely to achieve R0 resection. For borderline resectable PDAC, a modified, safer FOLFIRINOX regimen offers improved R0 resection rates and better prognosis. In contrast, patients with locally advanced PDAC may benefit more from novel vascular resection methods or enrollment in alternative clinical trials, regardless of neoadjuvant chemotherapy strategies.
